# Amplicon sequencing data profiling of rhizospheric microbiome associated with *Capsicum annuum* L. var. *Kulai* cultivated under organic farming

**DOI:** 10.1128/MRA.00745-23

**Published:** 2023-11-01

**Authors:** Munirah Tharek, Noriha Mat Amin

**Affiliations:** 1 Soil Science, Water and Fertilizer Research Centre, Malaysian Agricultural Research and Development Institute (MARDI), Serdang, Selangor, Malaysia; 2 Biotechnology and Nanotechnology Research Centre, Malaysian Agricultural Research and Development (MARDI), Serdang, Selangor, Malaysia; DOE Joint Genome Institute, Berkeley, California, USA

**Keywords:** rhizosphere microbiome, *Capsicum annuum* L. var *Kulai*

## Abstract

Data on the 16S rRNA and 18S rRNA amplicon sequencing from the rhizosphere of *Capsicum annuum* L. var. *Kulai* cultivated under organic farming are unveiled. The most dominant phyla for the 16S rRNA gene were *Actinobacteriota* and *Proteobacteria*. As for the 18S rRNA gene amplicon, *Charophyta* and *Fungi* were the major taxonomic groups.

## ANNOUNCEMENT

Chili Kulai (*Capsicum annuum* L. var. *Kulai*) is known as a high economic value crop due to its ability to produce good quality and high-yield fruits (1–3). Increased yields of Chili Kulai cultivated under organic farming practices have also been documented (3, 4). The cultivation of Chili Kulai under organic farming methods provides numerous benefits to both the environment and consumers. The implementation of organic farming practices reduces the production cost, improves the soil structure, and enhances the activities of soil microbes (4, 5). Considering the worldwide demand for organic agricultural products and the benefits of organic farming practices, the rhizosphere microbiome of Chili Kulai cultivated under organic farming methods was investigated. The amplicon sequence data of the rhizosphere microbiome of Chili Kulai cultivated under organic farming would provide ample information on the potential microbes involved in promoting plant growth, yield, and quality of organic chili.

In August 2021, soil surrounding the root region of three respective *Capsicum annuum* L. var. *Kulai* was collected from the Integrated Organic Farm at the Malaysian Research and Development Institute (MARDI) Serdang, Selangor, Malaysia (latitude 2.9917007°N, longitude 101.6958361°E). DNA was extracted from 250 mg of soil using the DNeasy PowerSoil Pro Kit (Qiagen, Germany) and pooled. The integrity, purity, and concentration of the DNA were determined by observation through 1% Tris-acetate-EDTA (TAE) gel electrophoresis and analyzed at absorbance ratios of A260/280 and A260/230 using a spectrophotometer (Implen NanoPhotometer N60/N50) and fluorometric quantification using iQuant Broad Range dsDNA Quantification kit. The purified metagenomic DNA was amplified using locus-specific sequence primers as follows: 16S V3–V4 [forward overhang: 5′ TCGTCGGCAGCGTCAGATGTGTATAAGAGACAG‐(CCTACGGGNGGCWGCAG); reverse overhang: 5′ GTCTCGTGGGCTCGGAGATGTGTATAAGAGACAG‐(GACTACHVGGGTATCTAATCC)] and 18S V4 [forward overhang: 5′ TCGTCGGCAGCGTCAGATGTGTATAAGAGACAG‐(GCGGTAATTCCAGCTCCAA); reverse overhang: 5′ GTCTCGTGGGCTCGGAGATGTGTATAAGAGACAG‐(AATCCRAGAATTTCACCTCT)]. The first part of library construction involved the amplification of the 16S rRNA/18S rRNA gene of the selected regions using locus-specific sequence primers. PCR reactions were performed with KOD Multi & Epi (Toyobo, Japan) (https://www.toyobo-global.com/seihin/xr/lifescience/support/manual/KME-101.pdf). The second part of library construction involved the attachment of dual indices to the amplicon PCR using Illumina Nextera XT Index Kit v2 following the manufacturer’s protocols. The quality of the libraries was measured using the Agilent Bioanalyzer 2100 System by Agilent DNA 1000 Kit and fluorometric quantification by Helixyte Green Quantifying Reagent. The libraries were normalized and pooled according to the protocol recommended by Illumina and sequenced using the MiSeq platform with 300-bp paired-end reads. Raw data were clustered into amplicon sequence variants using DADA2 V1.18 (6) and aligned against the SILVA v132 database. A total of 277,192 and 368,276 raw reads were extracted for 16S rRNA and 18S rRNA, respectively.

**Fig 1:**
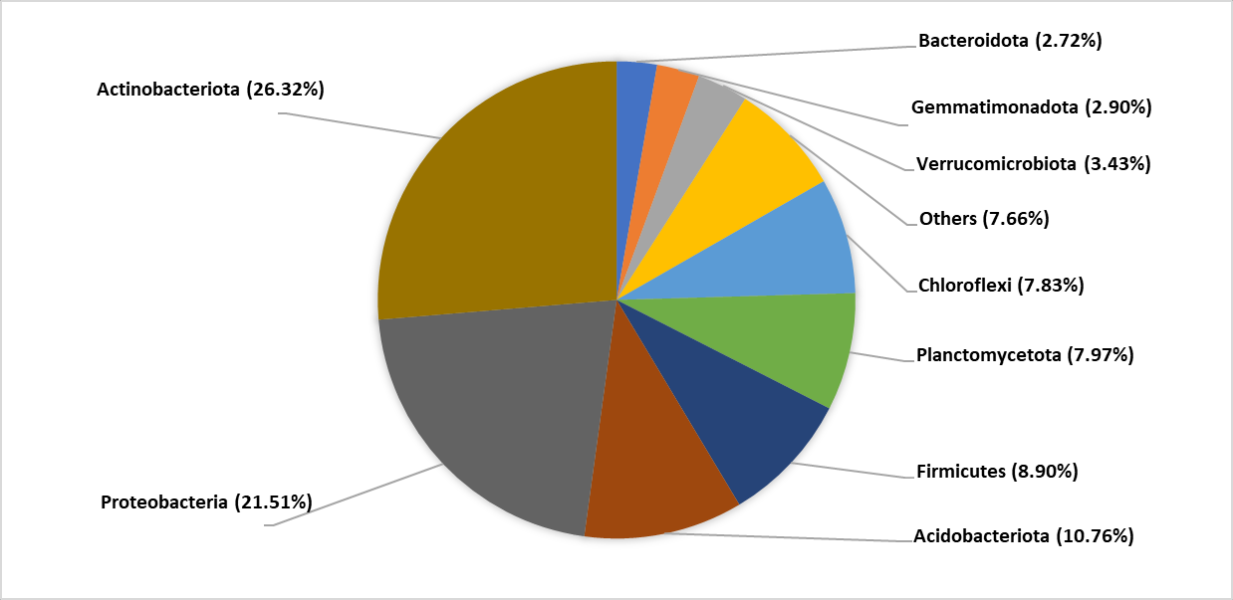
Percentage of key bacterial communities identified from the rhizosphere of *Capsicum annuum* L. var. *Kulai* cultivated under organic farming.

Analysis at the phylum level discovered *Actinobacteriota* (26.32%) and *Proteobacteria* (21.51%) as the most abundance phyla for 16S rRNA gene amplicon (Fig. 1). As for 18S rRNA gene amplicon, the major taxonomic groups were Charophyta (26.84%) and Fungi (26.14%) (https://doi.org/10.6084/m9.figshare.24030483.v1).

## Data Availability

The 16S rRNA and 18S rRNA gene amplicon sequencing data from this study have been deposited in the National Center for Biotechnology Information (NCBI) database under the Sequence Read Archive (SRA) accession numbers (SRX19970204 and SRX19970205) and the BioProject accession number PRJNA955764.
